# Prevalence and associated factors of birth injury among neonates admitted at neonatal intensive care unit (NICU) in governmental hospitals of Southwest Ethiopian people regional state, Ethiopia: A multicenteric cross-sectional study

**DOI:** 10.3389/fped.2022.1052396

**Published:** 2022-11-24

**Authors:** Alemayehu Sayih Belay, Ketemaw Negese, Gizachew Ayele Manaye, Shibihon Debebe

**Affiliations:** ^1^ Department of Nursing, Mizan Tepi University, College of Medicine and Health Sciences, Mizan Aman, Ethiopia; ^2^ Department of Midwifery, Mizan Tepi University, College of Medicine and Health Sciences, Mizan Aman, Ethiopia; ^3^ Department of Medical Laboratory Sciences, Mizan Tepi University, College of Medicine and Health Sciences, Mizan Aman, Ethiopia; ^4^ Department of Clinical Chemistry, Bahir Dar Health Sciences College, Bahir Dar, Ethiopia

**Keywords:** prevalence, neonatal birth injury, factors, hospitals, Ethiopia

## Abstract

**Introduction:**

Neonatal birth injury is the functional or structural damage of the new-born during child birth. Fetal related factors such as macrosomia, fetal height, fetal weight, and prematurity; maternal related factors such as overly young and old maternal age, parity, poor maternal health, and pelvic anomalies contribute to neonatal birth injury. Labor and delivery related factors including prolonged labor, fetal mal-presentation and mal-position, cesarean and instrumental deliveries also predispose the neonate to birth injury. This study was conducted to assess the prevalence and associated factors of birth injury among neonates admitted to the neonatal intensive care unit (NICU) in Governmental Hospitals of Southwest Ethiopia.

**Objective:**

To assess the prevalence and associated factors of birth injury among neonates admitted to the neonatal intensive care unit (NICU) in governmental hospitals in Southwest Ethiopia.

**Method:**

Hospital-based cross-sectional study design was implemented at Mizan-Tepi University Teaching Hospital, Bonga Gebretsadik Shawo General Hospital, and Tepi General Hospital. A total of 1,315 neonates were included in the study using systematic random sampling techniques. Data was entered using Epi-Data version 4.2 and exported to SPSS version 21 for analysis. Logistic regression analysis was conducted to see the association between the dependent and independent variable.

**Results:**

The prevalence of neonatal birth injury was 16.7%. Predictors such as primipara, no formal education, mothers with no antenatal care, and mothers whose occupational status were unemployed were 12.27, 2.52, 2.40, and 4.26 times more likely to develop neonatal birth injuries than their counterparts, respectively. Whereas, maternal age within the age range of 25–34 years, and neonates delivered *via* instrumental delivery were 6.68, and 2.81 times more likely to develop neonatal birth injury compared to those whose age was greater than 34 years and neonates delivered through Cesarean section, respectively.

**Conclusion:**

The magnitude of birth injury in the current study was significantly high. Primiparity, mothers with no history of antenatal care follow up, uneducated women, unemployed women, mode of delivery, and maternal age between 25 and 34 years were strong predictors associated with neonatal birth injury. Therefore, comprehensive maternal health care such as antenatal care follow up and health institution delivery should be promoted and well addressed to all reproductive age women and special attention should be given particularly to pregnant women in order to mitigate problems related to childbirth.

## Introduction

Neonatal birth injury is the functional or structural damage of the newborn during child birth (1). Birth injuries which are also known as birth traumas which result due to traumatic events such as traction and compression or health professional management errors of laboring mothers during labor/delivery (2–4).

Worldwide, an estimated 5.4 million children aged less than 5 years died. Of these deaths, 2.5 million (46%) occurred during the first 28 days of life (5). Severe birth trauma can be life-threatening, but early diagnosis and treatment increases the rate of survival (6).

Birth injury (BI), commonly occurs in the 2nd stage of labor as a result of normal forces of labor, contraction, twisting, and traction of neonates through the birth canal or as a sequel of obstetric intervention (7). It varies from self-limiting minor soft tissue injuries to severe, major life-threatening injuries that require early detection and intervention (8).

Neonatal birth injury was predisposed due to fetal related factors such as macrosomia, fetal height, fetal weight, and extreme prematurity and postdate; maternal related factors such as overly young and old maternal age, parity, poor maternal health, and pelvic anomalies contribute to neonatal birth injury (9–11). Labor and delivery related factors including prolonged labor, fetal mal-presentation and malposition, cesarean and instrumental deliveries also predispose the neonate for birth injury (7).

Neonates who suffer from injuries and diseases associated with lack of quality of care during child birth, lack of skilled health care provider, and lack of treatment immediately after birth and within the first days of life are at risk of dying within the first 28 days of life (called neonatal mortality). Preterm birth and intra-partum related complications such as birth asphyxia and lack of breathing at birth, infections, and birth defects caused the most neonatal deaths in 2017 (5).

Despite advances in obstetric care and prenatal diagnosis that play a pronounced role in decreasing the frequency of BI among newborn babies, BI is still recorded even in uncomplicated deliveries with the presence of optimal diagnostic tools (4).

The overall incidence of birth injuries in most developed countries has drastically declined due to improvements in obstetric care and prenatal diagnosis (12). However, in resource-limited settings including Ethiopia, the prevalence of neonatal injury remains unduly high (13). In Ethiopia, birth trauma and asphyxia could contribute for about 31.6% of neonatal deaths (14). Moreover, there was a paucity of data concerning neonatal birth injury, particularly in southwest Ethiopia. Thus, assessing the prevalence and factors associated with neonatal birth injury could provide evidence-based information and plays a major role in reducing neonatal deaths occurring during child birth. It will also provide a clue and will be used as a basis for future similar studies.

## Methods

### Study design, period and setting

A hospital-based multi-centered cross-sectional study was conducted from May 1st, 2021 to April 30th, 2022. These hospitals are Bonga G/tsadik Shawo General Hospital, Mizan Tepi University Teaching Hospital (MTUTH), and Tepi General Hospital. Currently, Mizan-Tepi University Teaching Hospital is expected to provide services to more than one million populations. Meanwhile, Bonga G/tsadik Shawo General Hospital and Tepi General Hospital are expected to provide care for more than 500,000 populations each. These hospitals provide health services and act as referral centers for other district primary hospitals and health centers. In 2020, the NICU in each hospital provided a service for a total of 1,200, 958, and 680 neonates in MTUTH, in Bonga Gebretsadik Shawo General Hospital and in Tepi General Hospital, respectively (Unpublished report).

### Study design and period

A multi-centered institutional based cross-sectional study design was conducted among neonates admitted in NICU in governmental hospitals of southwest Ethiopia from May 1st, 2021 to April 30th, 2022.

### Inclusion and exclusion criteria

#### Inclusion criteria

Neonates aged 0–28 days admitted to neonatal intensive care unit of governmental hospitals, southwest Ethiopia region.

#### Exclusion criteria

Neonates with known major fetal anomalies (tumors) and neonates with severely ill mothers were excluded from the study.

### Source and study population

#### Source population

All neonates admitted to NICU in all public hospitals in southwest Ethiopia were the source population.

#### Study population

All neonates admitted to NICU in selected public hospitals during the study period were the study population.

### Sample size determination

Sample size was determined using a single population proportion formula by considering the following parameters: *p* = 15.4% (15), *z* = 1.96, *d* = 0.02 and CI = 95%, where, *p* = proportion of birth injury; *z* = probability of normal distribution; *d* = margin of error, and CI=confidence level. Using 5% of the non-response rate, the total sample size (*n*) = 1,315. Proportional allocation to size was performed and representative samples were drawn from each hospital.

### Sampling procedure

**Figure 1:**
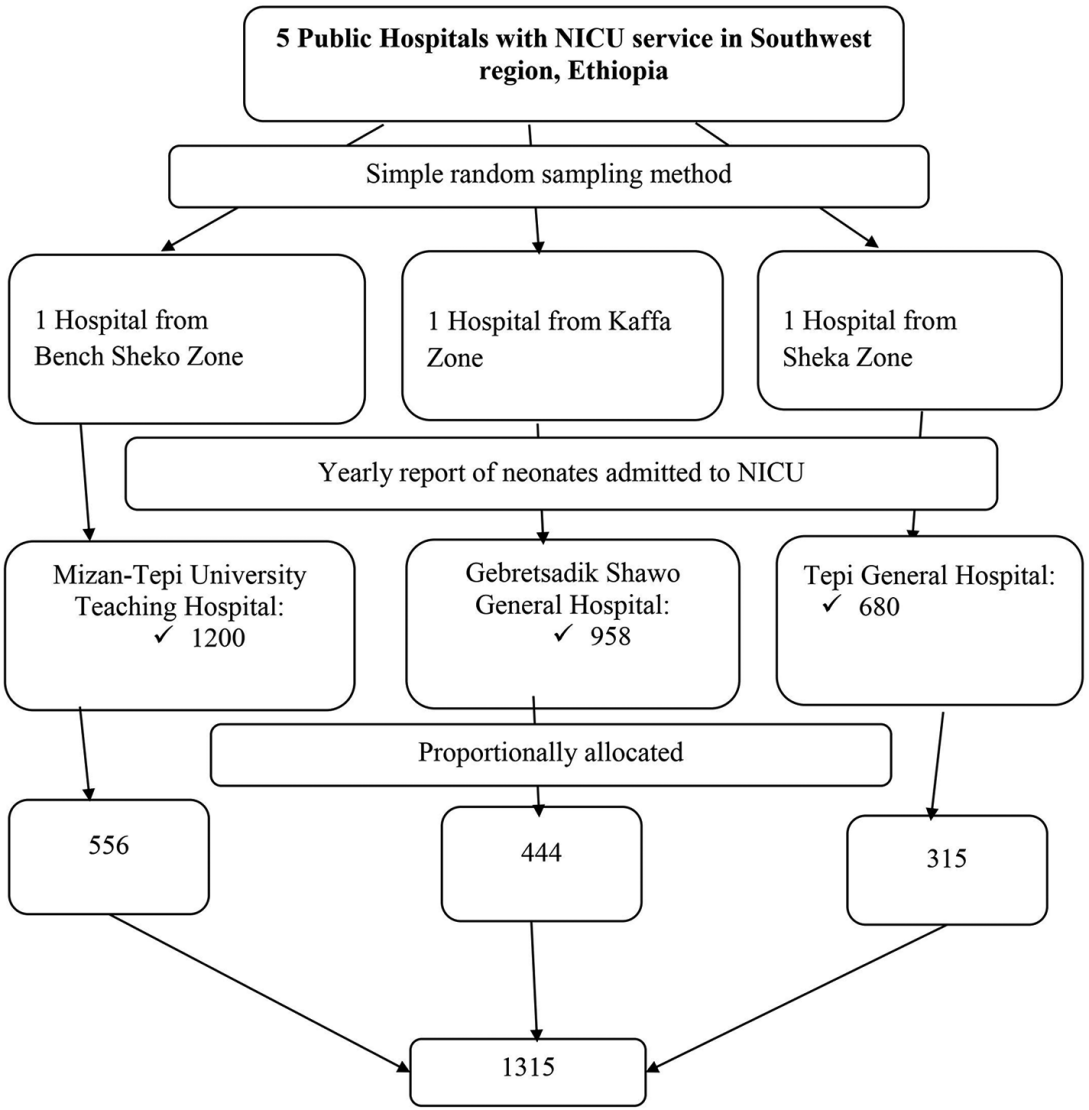
Schematic representation of the sampling procedure for neonates admitted at NICU ward in government hospitals located in Southwest Ethiopian people regional state, Ethiopia, 2022.

Multistage sampling method was used in this study. First, the number of hospitals with NICU services in the Southwest Region was determined. Accordingly, five public hospitals with NICU service, such as Bonga Gebretsadik Shawo General Hospital, Tepi General Hospital, MTUTH, Tercha General Hospital and Masha General Hospital were determined in the region. Then, three hospitals (Bonga Gebretsadik Shawo General Hospital, Tepi General Hospital and MTUTH were randomly selected using lottery method. The previous year annual report of neonatal admission to NICU in each selected hospital was taken. Then, using the total annual neonatal admissions at each selected hospital as a source population, the sample size was proportionally allocated to each selected public hospital and secondary sampling unit in which the neonates admitted to NICU were selected using systematic sampling method (2,838/1,315 = 2) based on the order of time of admission [Figure 1].

### Data collection tool, procedures and quality control

An interview administered structured questionnaire was adapted from reviewing different literature (16–18). The questionnaire was prepared and data was collected after formatted into three parts. Part-1: maternal socio-demographic characteristics, such as age, residence, religion, educational and occupational status; Part-2: obstetrical history, including history of antenatal care, mode of delivery, parity and source of referral; and Part-3: a review of records regarding neonatal birth trauma, anthropometric data (weight and length), and clinical data (APGAR score) was also conducted using the checklist. To maintain the quality of the results, three days of training was given to data collectors about the objective and relevance of the study, confidentiality issues, study participants' rights, consenting, and techniques of interview. Moreover, to ensure the quality of data, pretest, close supervision of data collection, and double-entry of data was done. Pre-test was conducted among 5% (66) neonates at Bachuma primary hospital for its accuracy and consistency. The data was then collected by three trained midwives and supervised by three trained MSc in clinical midwifery specialists.

### Measurements

#### Birth injury

It is defined as functional or structural damage to the body of a new-born following labor/delivery.

### Data processing and analysis

The data was checked for completeness and then the data was entered into Epi-Data Manager 4.2 and then transferred to the statistical package for social science (SPSS) version 21 for analysis. Descriptive statistics (frequency, percentage, mean and standard deviation) were used to describe socio-demographic variables, neonatal and obstetrical related variables, and clinical and anthropometric measurements.

Binary logistic regression analysis was performed to determine the significant association between each predictor (socio-demographic characteristics, neonatal and obstetrical related characteristics) and neonatal birth injury. Then, predictors with a *p*-value of <0.05 were included in a multivariable logistic regression model to identify the strong predictors significantly associated with neonatal birth injury.

The model fitness was checked with Hosmer and Lemeshow goodness of fit test at a *p*-value > of 0.05. Moreover, multicollinearity was checked using variance inflation factor (VIF) test and, the variance inflation factor (VIF) of less than 10 and tolerance of greater than 0.1 was considered as there was no multicollinearity. Finally, after adjusting for the confounding variables using multivariable logistic regression analysis, predictors with a *p*-value of <0.05 and a CI of 95% were considered as strong predictors associated with neonatal birth injury.

## Result

### Socio-demographic characteristics of the respondents

A total of 1,315 neonates were involved in the study, with a 100% response rate. The mean (±SD) age of the study participants was 31.26 ± (8.88) years, with most of them, 544(41.4%) were aged greater than 34 years of old. The majority, of 787(59.8%) of women were rural residents. More than two thirds, 963(73.2%) of the total respondents were orthodox religious followers. From the total participants, 804(61.1%) and 542(41.2%) were employed and didn't attend formal education, respectively [Table 1].

**Table 1:** Socio-demographic characteristics women who gave birth at governmental hospitals of Southwest Ethiopian people regional state, Ethiopia, 2022.

Variables	Category	Frequency	Percent (%)
Residence	Rural	787	59.8
	Urban	528	40.2
Religion	Orthodox	963	73.2
	Muslim	352	26.8
Maternal age in years	15–24	428	32.5
	25–34	343	26.1
	>34	544	41.4
	Mean ± (SD)	31.26 ± (8.88)	
Occupation	Employed	804	61.1
	Unemployed	511	38.9
Educational status	No formal education	542	41.2
	Formal education	773	58.8

### Magnitude and types of birth trauma

**Figure 2:**
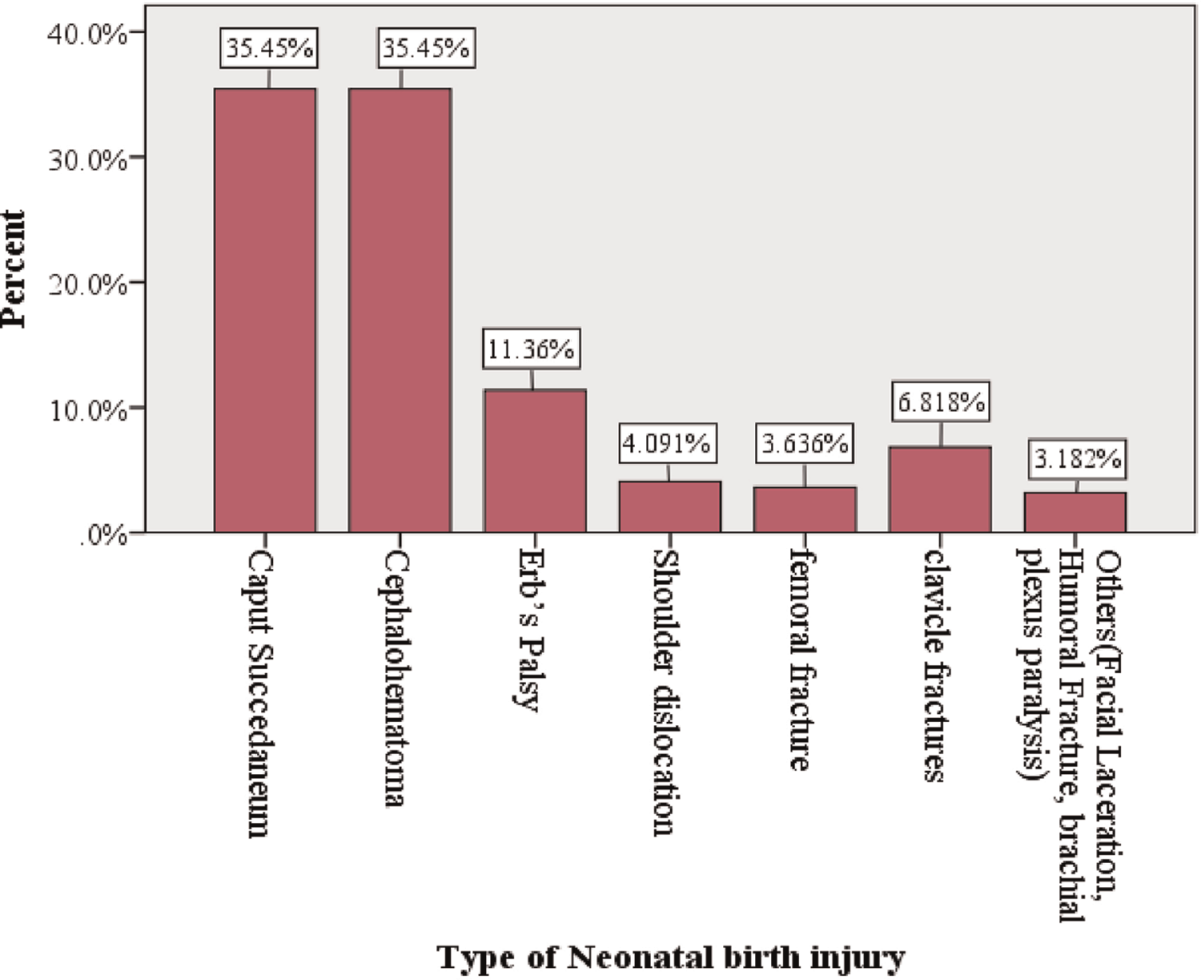
Type of neonatal birth injury among neonates admitted at NICU ward in government hospitals located in Southwest Ethiopian people regional state, Ethiopia, 2022.

From the total of 1,315 neonates admitted in NICU, 220 (16.7%) with a 9% CI of [16.7; 95% CI: 14.7, 18.7] have developed birth injury. Caput succedaneum and cephalhematoma were the major neonatal complications with an equal magnitude of 78 (35.4%), and followed by 11.36% and 6.82% of neonatal birth injury of Erbs's palsy and clavicle fracture, respectively [Figure 2].

### Obstetrical and neonatal characteristics

Regarding birth weight, majorities, 624 (47.5%) were below 2,500 g, 392 (29.8%) were within the range of 2,500–4,000 g, and 299 (22.7%) were above 4,000 g. Nearly half of the participants, 628 (47.8%) were preterm followed by term, 491 (37.3%), and post-term, 196 (14.9%). Out of the total number of study participants included in this study, almost all 1,267 (96.3%) were delivered at a health institution, and the remaining 48 (3.7%) of the participants were delivered at home.

Out of the total number of neonates in NICU, 504 (38.3%) of them developed complications. Of those who developed complication, 120 (23.8%) and 204 (15.5%) of the neonates develop anemia and neonatal sepsis, respectively.

Regarding obstetrical history, almost two thirds 999 (76%) neonates were delivered from mothers with a history of antenatal care follow up. More than half, 775 (58.9%) of neonates were delivered from multiparous mothers. Almost all of the mothers, 1,147 (87.2%) were referred from a health institution, while the remaining 168 (12.8%) were self-referred [Table 2].

**Table 2:** Obstetrical and neonatal characteristics women who gave birth at governmental hospitals of Southwest Ethiopian people regional state, Ethiopia, 2022.

Variables	Categories	Frequency	Percent (%)
Birth weight (g)	<2,500	624	47.5
	2,500–4,000	392	29.8
	>4,000	299	22.7
Birth height (cm)	Normal	564	42.9
	Abnormal	751	57.1
Gestational age (weeks)	Preterm	628	47.8
	Term	491	37.3
	Post term	196	14.9
Mode of delivery	Instrumental vaginal delivery	406	30.9
	Spontaneous vaginal delivery	756	57.5
	Caesarean section	153	11.6
Neonatal birth Injury	Yes	220	16.7
	No	1,095	83.3
Fetal presentation during labor	Vertex	1,171	89
	Non- vertex	144	11
Fetal position during labor	Normal	1,119	85.1
	Abnormal	196	14.9
Neonate develop complication	Yes	504	38.3
	No	811	61.7
Types of complication developed	Anemia	120	23.8
	Neonatal Sepsis	204	15.5
	Jaundice	180	13.7
Need of resuscitation	No	1,063	80.8
	Yes	252	19.2
Antenatal care (ANC)	No	316	24.0
	Yes	999	76.0
Parity	Primipara	540	41.1
	Multipara	775	58.9
Source of referral	Health institutions	1,147	87.2
	Self	168	12.8

### Factors associated with neonatal birth injury

Using bivariate logistic regression analysis, different variables such as gestational age, mode of delivery, antenatal care, educational status, occupational status, maternal age, birth weight, and parity were statistically associated with neonatal birth injury. But after adjusting for possible confounders using multivariate logistic regression analysis variables such as antenatal care, parity, educational status, occupational status, maternal age, and parity remained statistically significant predictors of neonatal birth injury.

Accordingly, women who were primipara (AOR: 12.27; 95% CI: 6.64–22.68), mothers with no formal education (AOR: 2.52; 95% CI: 1.46–4.37), mothers with no antenatal care (AOR: 2.40: 95% CI: 1.36–4.25), and mothers whose occupational status were unemployed (AOR: 4.26; 95% CI: 2.09–8.65), were 12.27, 2.52, 2.40, and 4.26 times more likely to develop neonatal birth injury than their counterparts, respectively. Whereas, maternal age with the age range of 25–34 years (AOR: 6.68; 95% CI: 2.85–15.65), and neonates delivered *via* instrumental delivery (AOR: 2.81; 95% CI: 1.45–5.45) were 6.68, and 2.81 times more likely to develop neonatal birth injury compared to those age were greater than 34 years and neonates delivered through cesarean section, respectively [Table 3].

**Table 3:** Factors associated with neonatal birth injury among women who gave birth in governmental hospitals of Southwest Ethiopian people regional state, Ethiopia, 2022.

Variables	Category	Birth injury		Logistic regression	
		Yes (%)	No (%)	COR (95% CI)	AOR (95% CI)
Maternal age in years	15–24	110 (25.7%)	318 (74.3%)	7.50 (4.72, 11.91)	1.97 (.99, 3.90)
	25–34	86 (25.1%)	257 (74.9%)	7.25 (4.50, 11.68)	6.68 (2.85, 15.65)^a^
	>34	24 (4.4%)	520 (95.6%)	1	1
Educational status	No formal education	130 (24.0%)	412 (76.0%)	2.40 (1.78, 3.22)	2.52 (1.46, 4.37)^a^
	Formal education	90 (11.6%)	683 (88.4%)	1	1
Occupational status	Employed	96 (11.9%)	708 (88.1%)	1	1
	Unemployed	124 (24.3%)	387 (75.7%)	2.36 (1.76, 3.17)	4.26 (2.09, 8.65)^a^
Antenatal care	Yes	115 (36.4%)	201 (63.6%)	1	1
	No	105 (10.5%)	894 (89.5%)	4.87 (3.59, 6.61)	2.40 (1.36, 4.25)^a^
Parity	Primiparous	172 (31.9%)	368 (68.1%)	7.08 (5.02, 9.98)	12.27 (6.64, 22.68)^a^
	Multiparous	48 (6.2%)	727 (93.8%)	1	1
Gestational age	Preterm	24 (3.8%)	604 (96.2%)	.023 (.02,.04)	.05 (.00, 2.67)
	Term	76 (15.5%)	415 (84.5%)	.12 (.08,.17)	.55 (.25, 1.19)
	Post term	120 (38.8%)	76 (61.2%)	1	1
Mode of delivery	Instrumental VD	184 (45.3%)	222 (54.7%)	3.40 (2.18, 5.30)	2.81 (1.45, 5.45)^a^
	SVD	6 (0.8%)	750 (99.2%)	.03(.01, .08)	.03(.01, .08)^a^
	Caesarean section	123 (80.4%)	30 (19.6%)	1	1
Birth weight	<2,500 g	24 (3.8%)	600 (96.7%)	.04(.03, .07)	.54(.01, 30.83)
	2,500–4,000 g	52 (13.3%)	340 (86.7%)	.17(.11, .24)	.66(.29, 1.49)
	>4,000 g	144 (48.2%)	155 (51.8%)	1	1

1: Reference category.

^a^ Significant variable at *p* value of <0.05, COR, Crude Odds Ratio; AOR, Adjusted Odds Ratio.

## Discussion

Worldwide, the incidence of neonatal birth trauma varies based on mode of delivery, fetal presentation, and fetal position, and is reported to be between 0.2 and 41.2 per 1,000 births (9, 19–21). In the current study, the prevalence of neonatal birth injury in southwest governmental hospitals was 16.7% with a 95% CI of [14.7, 18.7]. This finding was found to be higher compared to the studies conducted in Kashan City, Iran, 2.2% (17); millennium medical college, Addis Ababa, Ethiopia, 12.3% (22), and Special care baby unit in Maiduguri, North-Eastern Nigeria, 5.7% (23). This might be due to the decreased rate of instrumental delivery and a higher rate of cesarean sections preventing the incidence of birth trauma (17, 22). For instance, in this study, there were higher rate of instrumental delivery and lower incidence of cesarean sections. In contrast to these, the prevalence of neonatal birth injury in this study was found to be lower compared to the studies conducted in South-East Nigeria, 24.0% (24), and in Silte Zone, Ethiopia, 24.7% (25). The possible reason for this discrepancy might be due to the differences in the study setting, study design, infrastructure to the healthcare facilities, sample size, and the study period. For instance, four year prospective cohort study design was used in a study conducted in South-East Nigeria (24), but cross-sectional study design was used in this study. Therefore, many years prospective study design may contribute to the increment of the neonatal birth injury.

In this study, the mode of delivery was significantly associated with neonatal birth injuries. It was reported that neonates delivered *via* instrumental delivery (forceps/vacuum) are predisposed to develop injuries. This study was supported by studies conducted in Tikva, Israel, and Addis Ababa, Ethiopia, where caesarean delivery is a preventive factor for neonatal birth injury (9, 22). However, the study found that spontaneous vaginal delivery was a preventive factor for neonatal birth injuries relative to cesarean section. This might be due to variations in the study design, study settings, standard of obstetric care, and inappropriate use of the instrument during labor or inability of the medical profession to apply forceps/vacuum correctly.

Similarly, in this study, mothers with no history of antenatal care follow up were more likely to develop neonatal birth injuries. This is in agreement with a study conducted in Maiduguri, North-Eastern Nigeria (23). This might be due to the fact that mothers with no history of antenatal care might not have any health related information including choice in place of delivery, birth preparedness and complication readiness plan, and early identification of risky pregnancy due to medical and obstetrical problems. This in turn causes feto-maternal complications and increases the risk of neonatal birth injury (26, 27).

Neonates born to mothers who were not attending formal education and mothers who were unemployed had higher odds of developing neonatal birth injuries than their counterparts. This study finding is corroborated by studies conducted in Iran (28), Mumbi (21), and in Nigeria (23). This might be due to the reason that women with formal educational status increase their job opportunities and income generation, which in turn helps mothers to visit health institutions. Moreover, mothers from lower social classes were more vulnerable to develop more birth injuries (29).

In this study, parity was found to be a predisposing factor for neonatal birth injury. Other studies conducted in Kashan, Iran (17); southwest Nigeria (30), and in Chennai, India (31) also support this finding (17, 30, 31). This is due to the fact that neonates delivered from mothers with multiparty were less risky to develop neonatal birth injury than neonates delivered from women with primipara (4). This result is explained by the fact that the pelvic joints and muscles of the birth canal of primiparous women were tight compared to multiparious women and this may exert undue pressure on the fetal presenting part during the labor process.

Maternal age was also found to be significantly associated with neonatal birth injury. This study finding was also found to be consistent with the study conducted in low and middle income countries (32). This result might be explained by the fact that the pelvis and pelvic muscles of mothers with young age were contracted and tight enough, respectively, compared with mothers with old age, and this might predispose for neonatal birth injury.

### Strengths and limitations of this study

This study was conducted at three hospitals, which increased the probability of generalizability among neonates admitted in NICU. This study used a multivariate logistic regression analysis to control as many possible confounders as possible. Since this study was a cross-sectional study, it cannot show the cause and effect relationship of variables. In addition, some caput succedaneum and cephalohematoma may not cause severe illness but considered to be birth trauma in the study.

## Conclusion

The incidence of neonatal birth injury in southwest Ethiopia hospitals is higher (16.7%). Maternal age ranges from 25 to 34 years; neonates born from uneducated and unemployed mothers, prim parity, and mode of delivery (instrumental vaginal delivery) and mothers with no history of antenatal care follow-up during their pregnancy are statistically significant with neonatal birth injury. Thus, health education dissemination about antenatal care services for reducing neonatal birth injuries should be improved. Training should be provided to medical staff who attend laboring mothers regarding detecting abnormal progress in labor and also application of forceps and vacuum to assist vaginal delivery. Empowerment of women should be encouraged regarding education and health care services utilization. Primiparous women should also be well supervised during labor.

## Data Availability

The raw data supporting the conclusions of this article will be made available by the authors, without undue reservation.
